# Thermoregulatory, physiological, and intestinal responses to functional waters in heat-stressed rats

**DOI:** 10.14202/vetworld.2025.2761-2773

**Published:** 2025-09-18

**Authors:** Amani Al-Dawood, Raneem Al-Shalabi, Hosam Al-Tamimi, Raed Halalsheh

**Affiliations:** 1 Department of Applied Biology, College of Sciences, Tafila Technical University, Tafila 66110, Jordan; 2 Department of Nutrition and Food Technology, Faculty of Agriculture, Jordan University of Science and Technology, Irbid 22110, Jordan; 3 Department of Animal Production, Faculty of Agriculture, Jordan University of Science and Technology, Irbid 22110, Jordan; 4 Department of Medical Laboratory Sciences, Faculty of Applied Medical Sciences, The Hashemite University, Zarqa 13133, Jordan

**Keywords:** alkaline reduced water, functional waters, heat stress, intestinal morphology, ozonated water, thermoregulation, Zamzam water

## Abstract

**Background and Aim:**

Climate change and global warming have intensified the challenges of heat stress (HS) in mammals, compromising thermoregulation, hydration, and physiological stability. Functional waters such as alkaline reduced water (ALKA), Zamzam water (ZMZM), and ozonated water (OZON) have been proposed to provide therapeutic and protective benefits. However, limited research has explored their roles in thermoregulation under chronic HS. This study investigated the effects of ALKA, ZMZM, and OZON on thermophysiology, serum metabolites, and intestinal morphology in Wistar rats exposed to variable thermal climates.

**Materials and Methods:**

Seventy male Wistar rats (8 weeks old, 180–200 g) were randomized into five groups (n = 14): Control, distilled water (DIST), ALKA, ZMZM, and OZON. The experiment lasted 71 days, consisting of thermoneutral zone (TNZ; days 0–7), HS (32.3 ± 0.8°C; days 8–35), and a return to TNZ (days 36–71). Core body temperature (Tcore) was monitored using thermal microchips and loggers. Daily water intake, body weight, serum biochemical markers, and intestinal villi morphology were evaluated. Data were analyzed using repeated measures analysis of variance and general linear model procedures.

**Results:**

HS induced hyperthermia and increased daily water intake by 69.2% across all groups. ZMZM significantly reduced Tcore, especially at night, and increased water intake during HS. OZON consumption elevated water intake during TNZ, reduced serum creatinine, and enhanced alkaline phosphatase levels, while both OZON and DIST groups exhibited elevated antidiuretic hormone levels. ALKA intake significantly reduced serum sodium+ and chloride− levels under post-HS TNZ. Histological analysis revealed that ZMZM markedly increased villus length, width, and crypt depth in the duodenum, jejunum, and ileum, indicating enhanced intestinal absorptive capacity.

**Conclusion:**

ZMZM demonstrated superior thermoregulatory and intestinal benefits, supporting hydration, physiological recovery, and intestinal health during and after HS. OZON showed nephroprotective and metabolic modulation potential, while alkaline water contributed to electrolyte regulation. These findings highlight the potential of functional waters as supportive strategies against HS, warranting further translational studies in livestock and humans.

## INTRODUCTION

The World Health Organization has recently identified climate change and global warming as critical life-threatening challenges, largely due to the rising mortality rates associated with heat-related illnesses [1]. To cope with environmental fluctuations, the human body relies on a complex network of physiological responses and negative feedback mechanisms–collectively known as homeostasis–to maintain internal stability [2, 3]. Homoeothermic animals, in particular, regulate their core body temperature (Tcore) and peripheral blood flow to sustain a relatively constant body temperature under diverse environmental conditions [4]. Thermoregulation involves multiple processes, including behavioral adjustments and metabolic responses, which are mediated by alterations in calorigenic hormones [5].

Fluid homeostasis plays a central role in thermoregulation, compensating for water losses through panting and sweating [6]. Water metabolism ensures equilibrium between fluid intake and excretion [7]. During exposure to heat or exercise, fluid consumption must exceed sweat losses to maintain hydration. Importantly, beyond volume alone, water composition has been shown to significantly influence thermoregulation [8]. For instance, consuming plain water after exercise in hot conditions reduces plasma osmolality and sodium (Na) concentration, which in turn stimulates urine production and diminishes the drive to drink, ultimately delaying rehydration [9]. Conversely, intake of Na chloride-rich mineral water enhances water consumption, reduces urine output, and promotes fluid retention [10].

In recent years, increasing scientific attention has focused on functional waters for their potential health-promoting effects, particularly in mitigating oxidative stress, improving metabolic performance, and exhibiting antimicrobial properties. Among the most studied are alkaline reduced water (ALKA) [11], Zamzam water (ZMZM) [12], and ozonated water (OZON) [13].

Despite the growing concern about global warming and its impact on thermoregulation and fluid balance, limited attention has been given to the role of functional waters in mitigating heat stress (HS). While several studies have established that water composition influences rehydration efficiency and electrolyte balance [8–10], most investigations have focused on conventional or mineral waters, often neglecting functional water types with unique physicochemical properties. ALKA has been reported by García-Gómez *et a*l. [11] to improve hydration and antioxidant capacity, ZMZM is recognized for its distinct mineral profile and potential antimicrobial activity [12], and OZON has demonstrated oxidative modulation and vasodilatory properties [13]. However, existing research is fragmented, primarily limited to short-term biochemical evaluations or specific disease-related contexts, rather than comprehensive assessments under sustained HS conditions. Furthermore, evidence is particularly scarce on how these waters influence Tcore regulation, biochemical markers of hydration and stress, and intestinal histomorphology in animal models exposed to prolonged thermal challenges. This lack of integrative physiological, biochemical, and morphological insights creates a critical knowledge gap in understanding whether functional waters can serve as protective or supportive interventions in thermoregulation.

The present study was designed to address this gap by systematically investigating the thermophysiological and biochemical effects of three functional water types–ALKA, ZMZM, and OZON–compared with distilled water (DIST) and tap water (Control [CONT]) in heat-stressed Wistar rats. Specifically, the study aimed to:

1. Evaluate the influence of functional waters on Tcore dynamics during thermoneutral and HS conditions.
2. Assess their impact on water intake behavior and blood biochemical markers (including albumin, creatinine (CREAT), electrolytes, and hormones) as indicators of hydration, metabolic function, and stress response.
3. Examine the histomorphological characteristics of intestinal villi to determine structural adaptations associated with prolonged HS and different water types.

By integrating thermoregulatory, physiological, and morphological outcomes, this study provides a comprehensive assessment of functional waters as potential modulators of resilience to chronic HS. The findings are expected to contribute valuable insights into the translational application of functional waters in livestock and human health under climate-induced heat challenges.

## MATERIALS AND METHODS

### Ethical approval

All experiments were conducted in compliance with international regulations on animal welfare and Animal Research: Reporting of *In Vivo* Experiments guidelines. Approval was obtained from the Animal Care and Use Committee (ACUC; No. 5/2013), and all protocols were reviewed and authorized by the Research Deanship at Jordan University of Science and Technology (JUST).

### Study period and location

The study was conducted from June 2019 to November 2019 at the JUST animal house facility.

### Experimental animals and housing

This study included 70 male Wistar rats, aged 8 weeks and weighing 180–200 g, obtained from the animal colony at JUST. Experiments were conducted at an on-campus facility. Rats were housed individually in metallic cages (15 × 25 × 35 cm) to enable precise monitoring of feed intake, water intake, and body weight (BW). An electronic data logger (Onset U12-014, HOBO, Onset Computer Corporation, MA, USA) was suspended 1.8 m above the room center to continuously record ambient temperature, relative humidity, and illumination. The light cycle was maintained at 12 h dark (19:00–07:00) throughout the study. Rats were randomly assigned into five groups (n = 14/group) with similar BWs (pooled mean: 233.3 g; p = 0.9). The groups included: CONT, ALKA, OZON, ZMZM, and DIST.

These water types were selected due to their growing popularity and commercial availability, with many manufacturers making health claims about their benefits.

### Experimental design and thermal stress protocol

Before the trial, seven rats per group were randomly selected and fitted with injectable thermal microchips (LifeChip, USA), while the remaining underwent logger implantation surgery (described below). A 10-day recovery period was allowed before experiments began.

Animals were kept under standard conditions (12-h light/dark cycle, thermoneutral zone (TNZ) at 22.9°C ± 0.9°C, humidity 40%–60%) with ad libitum food and water access. The experiment lasted 71 days and comprised three phases:

1. Initial thermoneutral period (TNZ, days 0–7),
2. Chronic HS (32.3°C ± 0.8°C, days 8–35), and
3. Recovery thermoneutral period (days 36–71).

On days 34–35, blood samples were collected from microchip-implanted rats under anesthesia. Logger-implanted rats continued until day 71, after which blood was collected under anesthesia. Euthanasia was performed by cervical dislocation following ethical guidelines.

### Water preparation

- ALKA: Produced from tap water using a continuous electrolyzing apparatus (1BZSMO-0000053A2, Guangzhou Hibon Electronic Technology Co., China). pH 5.0–9.5; oxidation-reduction potential +400–−300 mV.
- ZMZM: Obtained directly from the Zamzam well without treatment.
- OZON: Prepared using a portable ozonator device (YL-A300N, CCAWJH Electronic Technology Co., China).
- DIST: Produced with a laboratory water distiller at JUST.
- CONT: Provided as standard tap water.

### Physiological and biometric measurements

Water intake and BW were monitored daily. Intake was measured as the difference between provided and refused amounts, using 150 mL rat drinking bottles with non-drip dispensers. Measurements were recorded once daily at 08:00 during TNZ and twice daily (08:00 and 17:00) during HS. Feed intake was recorded similarly, and BW was measured every 9–10 days to monitor growth.

### Tcore monitoring

Tcore was measured either manually with injectable thermal microchips or continuously at 1-h intervals using preprogrammed miniature loggers.

### Surgical procedure for logger implantation

Seven rats per group underwent intraperitoneal implantation of miniature thermochron loggers (iButton, USA) preprogrammed to record temperature at 0.0625°C resolution every 15 min. BW was recorded to optimize anesthetic dosage. An intraperitoneal cocktail of xylazine hydrochloride (0.25 mg/kg BW; Adwia, Jordan) and ketamine (0.4 mg/kg BW; Alfasan, Holland) was administered.

Deep sedation was verified by the absence of the paw-pinch reflex. The abdominal area was clipped, shaved, sanitized, and sterilized using alcohol and diluted iodine solution (1% free iodine, Al-Eiman, Jordan). Rats were placed in dorsal recumbency, covered with sterile drapes, and warmed to avoid anesthesia-induced hypothermia.

Post-operative care included prophylactic enrofloxacin (10 mg/kg BW; Hipra, Spain) for 14 days, starting 4 days pre-surgery, and analgesia with ibuprofen (10 mg/kg BW; Dar Al Dawa, Jordan) in drinking water (6.5 mL/L) for 5 days. Wound care included topical fucidin cream (20 mg/g Fusidic Acid; LEO, Denmark), diluted iodine, and hydrogen peroxide (10%) as required. Implantation followed procedures described by Al-Tamimi *et al*. [14].

### Blood sampling and biochemical analysis

On day 34 (end of HS), blood was collected from microchip-implanted rats, and on day 71 (end of TNZ), blood was collected from logger-implanted rats. Samples were drawn through cardiopuncture under anesthesia. Serum was separated by centrifugation (1,200 × *g*, 10 min) and stored at −20°C.

Serum CREAT, bilirubin, albumin, calcium, Na, chloride (Cl), and potassium were measured using commercial enzyme-linked immunosorbent assay kits (Wuhan Fine Biotech Co., Ltd., China). Additional analytes–including aldosterone (ALD), alanine transaminase (ALT), antidiuretic hormone (ADH), thioredoxin reductase 1, alkaline phosphatase (ALP), and malondialdehyde–were analyzed with specific kits from the same supplier.

Analyses were conducted in the Biotechnology Laboratory at JUST using a microplate reader (450 nm). Intra- and inter-assay coefficients of variation were <8% and <12%, respectively.

### Histological analysis of intestinal tissue

Duodenum, jejunum, and ileum samples (5 mm segments) were collected immediately post-mortem and fixed in 10% formalin for 3 days. Samples were dehydrated through graded alcohol concentrations (50%–100%), cleared in xylene, and embedded in paraffin. Sections (5 μm) were cut using a microtome and stained with hematoxylin–eosin.

Slides were deparaffinized in xylene, passed through descending alcohol concentrations (100%–50%), stained with hematoxylin, rinsed, stained with eosin, rinsed, and re-dehydrated with ascending alcohol concentrations.

Microscopic examination was conducted to measure villus height, width, and crypt depth. Slides were captured using Motic Image 2.0 software (Motic Co., China), and ImageJ [15] was used for morphometric analysis. At least five well-oriented villi per slide were evaluated.

### Statistical analysis

Tcore data were analyzed using split-plot analysis of variance [16] with mixed procedures (Proc Mixed) in SAS v9 (SAS Institute, NC, USA). The treatment was considered the main plot, and time × treatment as the subplot, with animals within treatment as random factors.

Serum biochemical and performance data were analyzed as a completely randomized design [17] using the general linear model procedure in SAS. Covariance structures (compound symmetry [CS] and autoregressive AR [1]) were compared, and the model with the highest Schwarz Bayesian criterion was selected [18].

All results are presented as least square means ± standard error, with statistical significance determined at p ≤ 0.05.

## RESULTS

### Thermoregulation and Tcore

**Figure 1:**
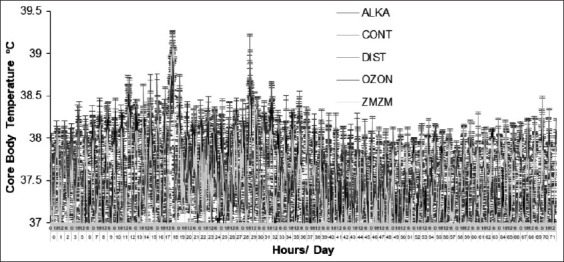
Mean (±1 SE) core body temperature of male Wistar rats fitted with miniature thermal loggers (n = 35) at 1-h intervals throughout the 71-day trial period. Rats were assigned to five treatment groups (n = 7) according to the type of water they drank: Reduced alkaline water (ALKA), control (CONT; consumed tap water), distilled water (DIST), ozonated water (OZON), or Zamzam water (ZMZM). The environmental management included an initial period of thermoneutral zone (TNZ); ambient temperature (Ta) = 22.9°C ± 0.9°C) between days 0 and 7, followed by 27 continuous days of chronic heat stress (Ta = 32.3°C ± 0.8°C), and ending with a second exposure to TNZ from day 36 to day 71. SE = Standard error.

**Figure 2:**
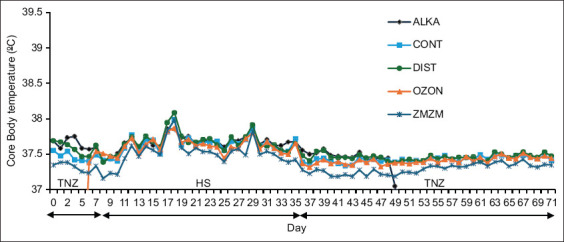
Mean (±1 SE) daily core body temperature of male Wistar rats fitted with miniature thermal loggers throughout the 71-day trial. Rats (n = 35) were assigned to five treatment groups (n = 7) according to the type of water consumed throughout the 71-day trial period: Reduced alkaline water (ALKA), control (CONT; consumed tap water), distilled water (DIST), ozonated water (OZON), or Zamzam water (ZMZM). The environmental management included an initial period of thermoneutral zone (TNZ); ambient temperature (Ta) = 22.9°C ± 0.9°C) between days 0 and 7, followed by 27 continuous days of chronic heat stress (Ta = 32.3°C ± 0.8°C), and a second exposure to TNZ from days 36 to 71. SE = Standard error.

A total of 1,704 Tcore readings per animal (59,640 readings for all 35 rats) were biotelemetrically recorded at 1-h intervals throughout the 71-day trial period (Figure 1). No complications, such as animal health-related abnormalities, resulted from any of the procedures. Moreover, no alterations in Tcore were observed among treatment groups throughout the trial period (Figure 2).

**Figure 3:**
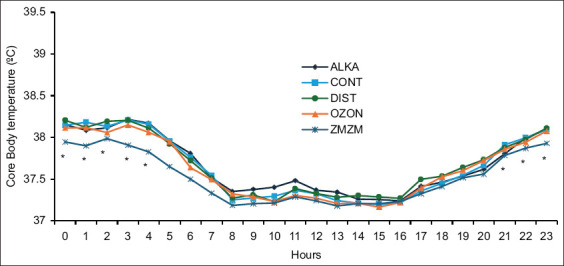
Mean (±1 SE) hourly core body temperature of rats fitted with miniature loggers and exposed to chronic heat stress (ambient temperature [Ta]) = 32.3°C ± 0.8°C) between days 8 and 35. Rats (n = 35) were assigned to five treatment groups (n = 7) according to the type of water drank: Reduced alkaline water (ALKA), control (CONT; consumed tap water), distilled water (DIST), ozonated water (OZON), or Zamzam water (ZMZM). An asterisk indicates a treatment-by-hour interaction (p = 0.000). SE = Standard error.

All groups exhibited a hyperthermia reaction, indicated by a significant rise in Tcore from TNZ to HS (p = 0.000). Interestingly, a treatment-by-hour interaction (p = 0.00) was observed throughout the trial, whereby the ZMZM group displayed lower Tcore than all other groups during the nighttime but not the daytime. A clear circadian rhythm (time effect; p = 0.000) was also evident in all rats, driven by the light cycle (nocturnal activity) and daily handling effects at 08:00–09:00 h and 17:00–18:00 h (Figure 3). Switching the ambient environment from HS back to TNZ resulted in the resumption of euthermia in all animals.

### Water intake

**Figure 4:**
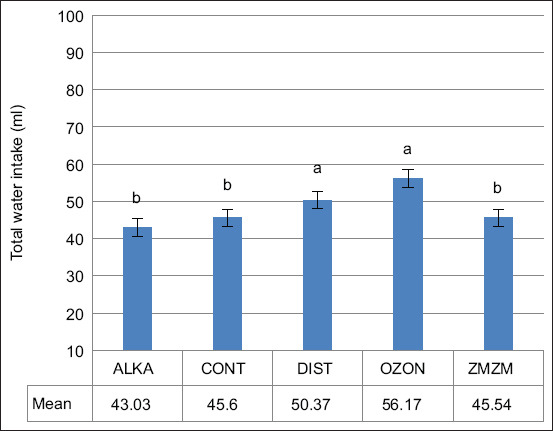
Mean (±1 SE) total water intake of male (mL) Wistar rats throughout the first thermoneutral zone period (TNZ); ambient temperature (Ta) = 22.9°C ± 0.9°C) between days 0 and 7. Rats (n = 70) were assigned to five treatment groups (n = 14) according to the type of water drank throughout the 71-day trial period: Reduced alkaline water (ALKA), control (CONT; consumed tap water), distilled water (DIST), ozonated water (OZON), or Zamzam water (ZMZM). Different superscript letters above bars indicate significant differences among the different treatments (p = 0.005). SE = Standard error.

Within the first TNZ, the OZON and DIST groups consumed significantly more water than the other three groups (p = 0.005), with mean values of ~57 mL (OZON) and ~51 mL (DIST), compared to ZMZM (~47 mL), CONT (~46 mL), and ALKA (~43 mL) (Figure 4).

**Figure 5:**
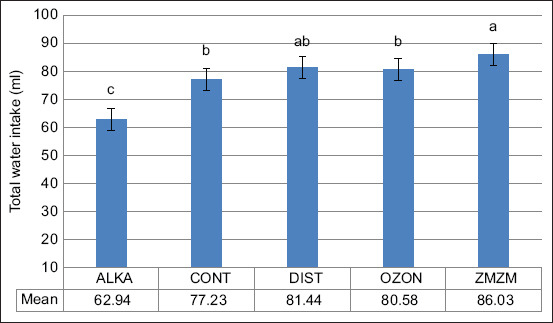
Mean (±1 SE) total water intake (mL) of male Wistar rats exposed to chronic heat stress (ambient temperature [Ta]) = 32.3°C ± 0.8°C) between days 8 and 35. Rats (n = 70) were assigned to five treatment groups (n = 14) according to the type of water they drank throughout the 71-day trial period: Reduced alkaline water (ALKA), control (CONT; consumed tap water), distilled water (DIST), ozonated water (OZON), or Zamzam water (ZMZM). Different superscript letters above bars indicate significant differences among the different treatments (p = 0.001). SE = Standard error.

**Figure 6:**
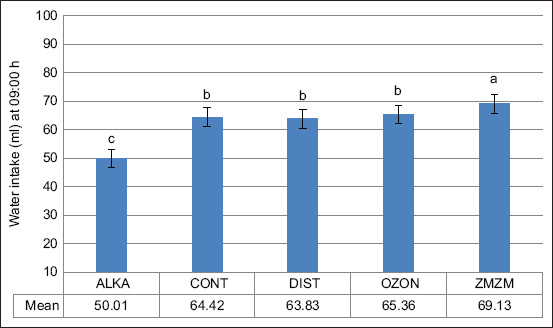
Mean (±1 SE) water intake (mL; measured at 09:00 h) of male Wistar rats exposed to chronic heat (ambient temperature [Ta]) = 32.3°C ± 0.8°C) between days 8 and 35. Rats (n = 70) were assigned to five treatment groups (n = 14) according to the type of water they drank throughout the 71-day trial period: Reduced alkaline water (ALKA), control (CONT; consumed tap water), distilled water (DIST), ozonated water (OZON), or water Zamzam (ZMZM). Different superscript letters above bars indicate significant differences among the different treatments (p = 0.001). SE = Standard error.

**Figure 7:**
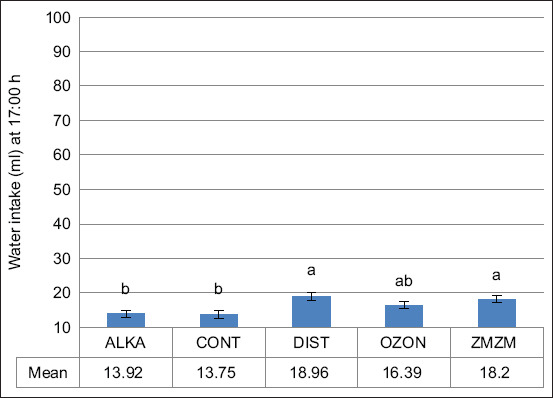
Mean (±1 SE) total water intake (mL; measured at 17:00 h) of male Wistar rats exposed to chronic heat stress (ambient temperature [Ta]) = 32.3°C ± 0.8°C) between days 8 and 35. Rats (n = 70) were assigned to five treatment groups (n = 14) according to the type of water they drank throughout the 71-day trial period: Reduced alkaline water (ALKA), control (CONT; consumed tap water), distilled water (DIST), ozonated water (OZON), or water Zamzam (ZMZM). Different superscript letters above bars indicate significant differences among the different treatments (p = 0.007). SE = Standard error.

**Figure 8:**
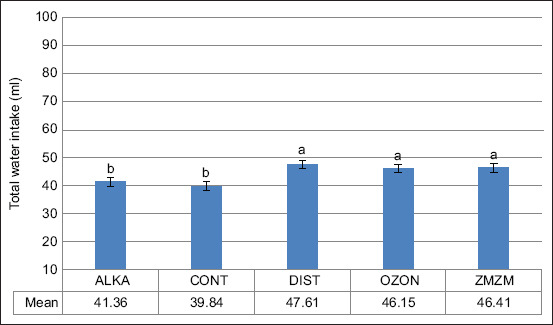
Mean (±1 SE) total water intake of male Wistar rats fitted with miniature thermal loggers throughout the second thermoneutral zone period; ambient temperature (Ta) = 22.9°C ± 0.9°C) between days 36 and 71. Rats (n = 35) were assigned to five treatment groups (n = 7) according to the type of water drank throughout the 71-day trial period: Reduced alkaline water (ALKA), control (CONT; consumed tap water), distilled water (DIST), ozonated water (OZON), or water Zamzam (ZMZM). Different superscript letters above bars indicate significant differences among the different treatments (p = 0.001). SE = Standard error.

Exposure to chronic heat resulted in a 69.2% increase in total daily water intake across all groups (p = 0.02) (Figure 5). The ZMZM group consumed significantly more water (~88 mL) than the other groups, whereas the ALKA group consumed less (~63 mL) (p = 0.001). Nighttime water consumption consistently exceeded daytime consumption (Figures 6 and 7). Upon returning to TNZ, water intake levels normalized in all animals (Figure 8).

Despite differences in water intake, the BW of rats did not vary significantly between treatment groups throughout the trial (p = 0.55).

### Biochemical indices of blood

As shown in Table 1, a significant treatment effect was observed for albumin, CREAT, ALP, and ADH. Rats consuming DIST water showed significantly lower albumin levels (p = 0.03) compared with those consuming ZMZM water. Conversely, OZON water mitigated the impact of HS on CREAT and ALP serum levels. Chronic heat increased CREAT levels; however, OZON water ingestion significantly reduced CREAT (p = 0.04), with no differences among the other groups.

**Table 1:** Blood biochemical indices of rats exposed to chronic heat stress and treated with different functional water types.

Parameters	Treatment					

	CONT	DIST	ALKA	OZON	ZMZM	SEM
Albumin (g/L)	31.6^ab^	30.46^b^	32.17^ab^	32.48^ab^	33.53^a^	1.05
Sodium (mmol/L)	123.49	122.11	130.20	122.27	130.04	4.67
Potassium (mmol/L)	4.22	4.11	4.34	3.85	3.99	0.40
Chloride (mmol/L)	102.19	98.20	106.15	104.28	106.02	3.90
Calcium (mg/dL)	10.17	9.33	9.96	10.24	10.23	0.52
Bilirubin (mg/dL)	0.08	0.06	0.12	0.11	0.14	0.04
CREAT (mg/dL)	0.72^a^	0.75^a^	0.85^a^	0.47^b^	0.76^a^	0.07
ALT (ng/mL)	130.69	121.68	133.70	147.43	160.82	22.16
ALP (ng/mL)	169.02^b^	166.89^b^	172.91^b^	182.57^a^	174.17^b^	3.29
IntThior (ng/mL)	4.12	3.54	3.98	3.57	3.834	0.38
Aldosterone (pg/mL)	60.28	55.07	55.01	66.01	42.07	14.75
MDA (ng/mL)	33.23	34.81	33.90	33.87	30.30	3.80
ADH (pg/mL)	2.41^b^	5.93^a^	2.77^b^	4.99^a^	2.12^b^	0.59

Rats (n = 25) were assigned to five treatment groups (n = 5) according to the type of water drank: Reduced alkaline water (ALKA), control (CONT; consumed tap water), distilled water (DIST), ozonated water (OZON), or Zamzam water (ZMZM). The environmental management included an initial period of thermoneutral zone (TNZ; ambient temperature (Ta)) = 22.9°C ± 0.9°C) between days 0 and 7, followed by 27 continuous days of chronic heat stress (Ta = 32.3°C ± 0.8°C). SEM = Standard error of the means, CREAT = Creatinine; ALT = Alanine transaminase, ALP = Alkaline phosphatase, MDA = Malonaldehyde, ADH = Antidiuretic hormone. Different superscript letters indicate significant differences among the different treatments within the same parameter (p = 0.02–0.04)

Regarding ALP, the OZON group exhibited a significantly higher level compared with other groups (p = 0.04). Chronic heat also appeared to increase ADH secretion due to dehydration. In this study, the DIST and OZON groups had significantly higher ADH levels than the other three water groups (p = 0.02). For other parameters, as shown in Table 1, no significant differences (p > 0.05) were detected between groups.

A significant treatment effect (p = 0.04) was also evident for Na, Cl, and ALT (Table 2). Interestingly, rats consuming ALKA water, despite being mineral-rich, showed lower Na levels compared with the CONT and ZMZM groups. Similarly, ALKA rats had lower Cl levels compared with CONT, though not significantly different from the other groups.

**Table 2:** Blood biochemical indices of rats maintained under thermoneutral zone and treated with different functional water types after exposure to chronic heat stress.

Parameters	Treatment					

	CONT	DIST	ALKA	OZON	ZMZM	SEM
Albumin (g/L)	33.18	33.46	33.71	32.68	32.47	1.08
Sodium (mmol/L)	138.15^a^	132.79^bc^	131.86^c^	132.83^bc^	136.46^ab^	1.29
Potassium (mmol/L)	4.98	4.80	4.86	4.90	5.03	0.10
Chloride (mmol/L)	106.55^a^	103.10^ab^	102.58^b^	103.45^ab^	104.17^ab^	1.12
Calicum (mg/dL)	10.10	10.25	10.55	9.74	10.22	0.27
Bilirubin (mg/dL)	0.10	0.07	0.10	0.11	0.17	0.036
CREAT (mg/dL)	0.80	0.83	0.82	0.77	0.81	0.10
ALT (ng/mL)	138.04^ab^	122.02^b^	142.12^ab^	154.14^a^	155.41^a^	9.08
ALP (ng/mL)	168.79	150.27	160.75	169.67	159.94	6.86
IntThior (ng/mL)	3.83	4.08	4.30	4.28	4.08	0.26
Aldosterone (pg/mL)	44.93	47.76	52.37	56.99	46.43	0.00
MDA (ng/mL)	32.29	31.83	33.54	36.75	32.61	3.80
ADH (pg/mL)	2.95	2.12	3.79	3.16	2.35	1.11

Rats (n = 25) were assigned to five treatment groups (n = 5) according to the type of water drank: Reduced alkaline water (ALKA), control (CONT; consumed tap water), distilled water (DIST), ozonated water (OZON), or Zamzam water (ZMZM). The environmental management included an initial period of thermoneutral zone (TNZ; ambient temperature [Ta]) = 22.9°C ± 0.9°C) between days 0 and 7, followed by 27 continuous days of chronic heat stress (Ta = 32.3°C ± 0.8°C), and a second exposure to TNZ from days 36 to 71. SEM = Standard error of the means, CREAT = Creatinine, ALT = Alanine transaminase, ALP = Alkaline phosphatase, MDA = Malonaldehyde, ADH = Antidiuretic hormone. Different superscript letters indicate significant differences among the different treatments within the same parameter (p = 0.04)

Switching the environment back to TNZ did not reverse the HS-induced hepatic effects in CONT, ALKA, OZON, and ZMZM groups. However, the DIST group showed lower ALT levels compared with OZON and ZMZM. For all other parameters, no significant differences (p > 0.05) were found between water groups (Table 2).

### Histological analysis

As shown in Table 3, ZMZM water significantly increased the length and width of duodenum, jejunum, and ileum villi (p = 0.000), resulting in greater villus surface area compared with other treatments. OZON water significantly increased duodenal villus width (p = 0.04) and ileal villus length (p = 0.000), with no effect on ileal width. CONT water significantly increased jejunum villus length (p = 0.001), but not villus width. DIST water significantly increased jejunum villus width (p = 0.000), with no effect on length.

**Table 3:** Histological analysis of the intestinal tissue of rats maintained under thermoneutral zone and treated with different functional water types after exposure to chronic heat stress.

Treatment	Parameters							

	Length (µm)	SEM	Width (µm)	SEM	Crypt depth (µm)	SEM	Surface area (mm^2^)	SEM
Duodenum								
ALKA	0.1473^b^	0.003	0.1286^c^	0.000	0.026^c^	0.001	0.006^c^	0.000
CONT	0.1231^c^	0.004	0.0166^b^	0.001	0.026^c^	0.001	0.006^c^	0.000
DIST	0.1516^b^	0.003	0.0167^b^	0.000	0.030^b^	0.001	0.007^b^	0.000
OZON	0.1318^c^	0.003	0.0194^a^	0.000	0.030^b^	0.001	0.008^b^	0.000
ZMZM	0.1863^a^	0.003	0.0197^a^	0.000	0.039^a^	0.001	0.010^a^	0.000
Jejunum								
ALKA	0.116^b^	0.000	0.012^c^	0.000	0.021^c^	0.001	0.004^c^	0.000
CONT	0.160^a^	0.000	0.015^b^	0.000	0.025^b^	0.001	0.007^b^	0.000
DIST	0.123^b^	0.000	0.018^a^	0.000	0.025^b^	0.001	0.007^b^	0.000
OZON	0.117^b^	0.000	0.018^a^	0.000	0.031^a^	0.001	0.007^b^	0.000
ZMZM	0.160^a^	0.000	0.018^a^	0.000	0.031^a^	0.001	0.009^a^	0.000
Ileum								
ALKA	0.145^c^	0.006	0.027^c^	0.003	0.016^b^	0.010	0.013^d^	0.002
CONT	0.190^b^	0.005	0.0402^b^	0.003	0.044^ac^	0.009	0.026^c^	0.001
DIST	0.114^d^	0.006	0.035^bc^	0.003	0.025^bc^	0.010	0.014^dc^	0.002
OZON	0.251^a^	0.006	0.044^b^	0.003	0.043^ab^	0.010	0.035^b^	0.002
ZMZM	0.188^b^	0.006	0.107^a^	0.003	0.059^a^	0.010	0.063^a^	0.002

Rats (n = 25) were assigned to five treatment groups (n = 5) according to the type of water drank: Reduced alkaline water (ALKA), control (CONT; consumed tap water), distilled water (DIST), ozonated water (OZON), or Zamzam water (ZMZM). The environmental management included an initial period of thermoneutral zone (TNZ; ambient temperature (Ta)) = 22.9°C ± 0.9°C) between days 0 and 7, followed by 27 continuous days of chronic heat stress (Ta = 32.3°C ± 0.8°C), and a second exposure to TNZ from days 36 to 71. SEM = Standard error of the means. Different superscript letters indicate significant differences among the different treatments within the same parameter and the same intestinal tissue (p = 0.000–0.04)

In contrast, ALKA water did not significantly affect any villus parameter. Importantly, ZMZM water also significantly increased crypt depth of the duodenum, jejunum, and ileum villi compared with the other water types (p = 0.001).

## DISCUSSION

### Thermoregulation and HS

The upper critical temperature in rats is estimated to be 31°C–32°C [19]. The normal body temperature of rats is approximately 37°C [20], while a Tcore of 40°C is considered a critical threshold for morbidity and mortality [21]. In the present study, all animals exhibited a hyperthermic reaction when exposed to chronic heat (Figure 2). This increase in Tcore was accompanied by an increase in total daily water intake, indicating a strong compensatory trend. However, the measured daily water intake does not necessarily represent the total amount ingested, since rats often use water for cutaneous evaporative cooling by spreading it on their bodies, particularly on their elbows, due to their inability to sweat. Thus, rats exposed to HS frequently demonstrate water-playing behavior.

Mammals also exhibit a circadian rhythm, a daily cycle of biological activity influenced by environmental fluctuations such as light–dark transitions. In this study, Tcore showed a biphasic pattern, with a gradual rise before 1900 h followed by a rebound after 0700 h (Figure 3). In addition, simple routine handling, feeding, watering, and cage cleaning elevated Tcore [22]. When comparing measurement techniques, large discrepancies were observed between Desetron thermal mcrochips (LifeChip, Desetron Fearing, USA) and iButtons (Maxim Integrated, iButton Devices, USA). The Desetron method required manual scanning and animal handling, which may itself affect Tcore, whereas iButtons provided more reliable data by automatically recording at 1-h intervals without handling. These findings agree with Gordon’s results [19], who reported circadian rhythm effects on Tcore in rats.

The nocturnal nature of rats explains their higher nighttime water intake compared to daytime (Figures 6 and 7). During the TNZ, the OZON group consumed significantly more water than the ALKA, CONT, and ZMZM groups, though not significantly different from the DIST group (Figure 4). This may be attributed to OZON water enhancing oxidation processes, stimulating cellular respiration and metabolic rate [23]. Ozone is also known for vasodilatory effects [24], and prolonged OZON consumption may increase diuretic responses, leading to a hyperosmolar state that triggers thirst [25].

Rats also drank more DIST water compared to CONT, ZMZM, and ALKA groups (Figure 4). This could be explained by changes in saliva pH (6.2–7.6), which activate taste nerves and stimulate drinking [26, 27]. Furthermore, chronic DIST water consumption may induce osmotic stress by altering ionic and molecular composition of extracellular and intracellular fluids [28], which activates osmoreceptors and enhances thirst sensation [29]. Under HS, the ZMZM group consumed significantly more water than CONT, OZON, and ALKA groups, with levels comparable to the DIST group (Figure 5). This finding supports El-Zaiat [30], who reported that the high calcium content of ZMZM water enhances its capacity to satisfy both thirst and hunger.

Interestingly, ZMZM water resulted in the lowest Tcore compared with other groups (Figure 2), suggesting superior thermoregulatory efficacy. Rodents typically dissipate heat through panting or salivation [31]. Salivary secretion is controlled by the autonomic nervous system but is also dependent on the water permeability of acinar cells [32], which are probable sites of aquaporin expression [33, 34]. ZMZM water has been shown to stimulate aquaporin expression in endometrial tissue [35], and a similar effect in salivary glands could enhance secretion under HS, improving thermoregulation. Further studies are required to validate this hypothesis.

The intestine represents the largest interface between the body and the external environment [36]. In this study, rats drinking ZMZM water had the largest intestinal surface area (Table 3), which may explain their significant nighttime reduction in Tcore compared to other groups. Conversely, the ALKA group consumed less water, likely due to its high electrolyte content enhancing hydration potential. This aligns with Li *et al*. [37], who reported that ALKA water improves absorption and retention.

### Blood biochemical indices

In this trial, ZMZM water significantly increased serum albumin levels, whereas DIST water failed to counteract the effects of HS, leading to reduced albumin concentrations. This finding agrees with Mahmoud *et al*. [38], who observed higher albumin in mice consuming ZMZM and CONT water compared to those drinking DIST water. ZMZM is known for its antioxidant capacity, acting as a potent reactive oxygen species (ROS) scavenger [11].

OZON water prevented the elevation of serum CREAT induced by chronic HS. This is consistent with Delgadillo-Valero *et al*. [39], who reported reduced CREAT and urea in rats treated with ozone therapy following acetaminophen-induced nephrotoxicity, and with Borrego *et al*. [40], who demonstrated ozone’s protective effect against cisplatin-induced nephrotoxicity. Ozone therapy is thought to mitigate oxidative damage by inhibiting the xanthine/xanthine oxidase pathway, thereby decreasing ROS production after reperfusion, while enhancing endogenous antioxidant systems [41, 42] and improving circulation and oxygen metabolism [43].

Elevated ALP activity typically indicates tissue damage, particularly in the liver and muscles [44]. In this study, all groups except OZON showed increased ALP levels. The protective effect of OZON water may be linked to its antioxidant properties, which attenuate chronic HS damage. Severe dehydration induced by HS can stimulate the renin–angiotensin– ALD system, leading to increased ADH secretion [45]. Hydration status depends on fluid type, composition, and mineral content [46]. Our findings indicate that water with alkalizing properties, such as ZMZM and ALKA, significantly improved hydration under HS. These findings support the findings of Li *et al*. [37], who reported improved acid–base balance and hydration with ALKA water in athletes, and Chiron *et al*. [47], who found bicarbonate-rich water enhanced post-exercise acid–base balance.

Rats consuming ALKA water, rich in electrolytes, showed lower serum Na and Cl levels compared to ZMZM and CONT groups (Table 2). The higher intestinal surface area observed with ZMZM water (Table 3) may account for greater mineral absorption [36]. ZMZM water contains high concentrations of Na, Cl, and trace elements [48], while the elevated Cl in the CONT group likely reflects chlorination processes in drinking water [49]. Despite the antioxidant benefits of ZMZM [12] and ALKA [50], ZMZM water unexpectedly increased ALT levels, in contrast to Elnour *et al*. [51] and Al-Doghaither *et al*. [52], who reported hepatoprotective effects. Similarly, the OZON group showed elevated ALT, which contradicts the findings of EroĞlu *et al*. [53], who reported that ozone reduced ALT. These discrepancies may be due to prolonged exposure in the present study.

### Histological analysis

Stress can alter intestinal microbiota and facilitate pathogen colonization, negatively affecting intestinal morphology [54]. Stress exposure also induces oxidative stress, leading to intestinal injury [55]. ZMZM water, with its antioxidant [12] and antimicrobial properties [56], may counteract these effects. Its antimicrobial action may be linked to its nitrate, fluoride, silver, and copper content [57–59]. These properties likely explain the significant improvement in histological parameters in the ZMZM group (Table 2).

Crypt depth reflects tissue turnover, with greater depth indicating faster regeneration and higher metabolic demand [60]. Starvation reduces villus height and crypt depth due to slowed cell production [61–63], while HS and dehydration similarly decrease food intake [64]. In this trial, ZMZM water significantly increased crypt depth, likely reflecting better hydration and food intake, supported by higher water consumption and stable Tcore in this group (Figures 3 and 5). In contrast, reduced villus height and crypt depth in ALKA, CONT, DIST, and OZON groups suggest temporary starvation during chronic HS.

## CONCLUSION

This study demonstrated that chronic HS induced hyperthermia in rats, accompanied by significant changes in water intake, biochemical indices, and intestinal morphology depending on the type of water consumed. Among the tested groups, ZMZM water provided the most notable thermoregulatory benefits, resulting in the lowest Tcore values, improved hydration, elevated serum albumin levels, and enhanced intestinal morphology compared with other water types. OZON water offered partial protection by attenuating serum CREAT elevation, while ALKA water supported hydration due to its high electrolyte content. In contrast, DIST water stimulated higher water intake but was associated with reduced albumin levels and osmotic stress.

These findings highlight the practical importance of water composition in mitigating the physiological consequences of HS. The results suggest that ZMZM and ALKA water, in particular, may play a valuable role in improving hydration, maintaining metabolic function, and supporting intestinal health in animals exposed to elevated environmental temperatures. Such insights have potential implications for laboratory research, livestock management, and animal welfare in regions experiencing frequent heat waves.

A major strength of this work lies in its integrative approach, combining thermoregulation, water intake behavior, biochemical markers, and histological analysis to provide a comprehensive understanding of adaptive responses. Nevertheless, limitations include its restriction to a single species under controlled laboratory conditions and the short experimental duration, which does not fully address long-term or cross-species effects. The unexpected elevation of ALT in ZMZM and OZON groups also warrants further mechanistic investigation.

Future research should explore the molecular mechanisms underlying aquaporin regulation, salivary gland function, and intestinal adaptation in response to different water compositions. Extending the findings to other species, particularly livestock, and conducting long-term safety assessments would strengthen translational applicability.

In conclusion, this study emphasizes the importance of water type in thermoregulation and physiological resilience under chronic HS. ZMZM water, in particular, appears to offer unique thermoprotective and antioxidant benefits. These findings contribute to a broader understanding of environmental stress physiology and may inform practical strategies to improve animal health, productivity, and welfare under conditions of global climate change.

## AUTHORS’ CONTRIBUTIONS

AA: Conceptualization, methodology, and preparation of original draft, review, and editing. RA: Investigation and preparation of original draft. HA: Conceptualization, formal analysis, methodology, and preparation of the original draft. RH: Conceptualization and methodology. All authors have read and approved the final manuscript.
